# A retrospective study: exploring preoperative hyponatremia in elderly patients with hip fractures

**DOI:** 10.1186/s13018-024-04643-w

**Published:** 2024-03-16

**Authors:** Li-Tao Shi, Zhen Feng, Cui-Min Zhu

**Affiliations:** 1 Trauma Department of Orthopedics, The Affiliated Hospital of Chengde Medical University, No. 36 of Nanyingzi Street, Shuangqiao District, Chengde, 067000 China; 2 Department of Ultrasound, Longhua County Guo Jia Tun Central Hospital, Chengde, 067000 China

**Keywords:** Femoral neck fracture, Hip fracture, Hyponatremia, Intertrochanteric femur fracture

## Abstract

**Background:**

This research aims to examine the frequency, age-related distribution, and intensity of preoperative hyponatremia among elderly individuals with hip fractures. This study aims to provide valuable insights into the diagnosis of preoperative hyponatremia in this patient population.

**Methods:**

This research involved the analysis of clinical data obtained from 419 elderly individuals with hip fractures (referred to as the fracture group) and 166 elderly individuals undergoing routine health examinations (designated as the control group). A comprehensive comparison was conducted, examining baseline characteristics such as age, gender, and comorbidities between these two groups. We further investigated variations in the incidence rate of hyponatremia, age distribution, and the severity of hyponatremia. Additionally, a subgroup analysis compared patients with femoral neck fractures to those with intertrochanteric femur fractures, specifically examining the incidence rate and severity of hyponatremia in these distinct fracture types.

**Results:**

The incidence of cerebrovascular disease was found to be higher in the fracture group as compared to the control group in our research. Nevertheless, no significant differences in general health and other comorbidities were observed between the two groups. Notably, the fracture group exhibited a greater preoperative prevalence of hyponatremia, with its severity increasing with age. Furthermore, among elderly patients with intertrochanteric femur fractures, the incidence of preoperative hyponatremia was not only higher but also more severe when compared to those with femoral neck fractures.

**Conclusion:**

Elderly individuals experiencing hip fractures exhibit a notable prevalence of preoperative hyponatremia, predominantly mild to moderate, with an escalating occurrence linked to advancing age. This phenomenon is especially conspicuous among patients with intertrochanteric fractures, warranting dedicated clinical scrutiny. The administration of sodium supplementation is advisable for the geriatric demographic as deemed necessary. Addressing hyponatremia becomes crucial, as it may play a role in the etiology of hip fractures in the elderly, and rectifying this electrolyte imbalance could potentially serve as a preventive measure against such fractures.

## Introduction

Hip fractures, encompassing femoral neck fractures and intertrochanteric femur fractures [1, 2], are predominantly observed in the elderly population, and surgeon is the main treatment option [3, 4]. Among the ongoing demographic aging trends, there is a continual increase in the annual incidence of hip fractures among the elderly population [5, 6]. The incidence of perioperative complications in elderly patients with hip fracture is high, some complications are closely related to the prognosis of patients, electrolyte disturbance is one of the most common complications [7–9]. Among the elderly, hyponatremia stands out as the most prevalent electrolyte imbalance [10–12], frequently presenting as a preoperative complication in patients with hip fracture [13, 14]. Failure to address preoperative hyponatremia in a timely manner not only results in delays in surgical interventions but may also precipitate severe cardiovascular and cerebrovascular events [15–18]. While recent research [19–21] has primarily explored the relationship between hyponatremia, fractures, and osteoporosis, there is a notable dearth of studies specifically addressing the prevalence of preoperative hyponatremia in elderly patients with hip fractures(and the other research illustrates differences in the prevalence of hyponatremia by sex and fracture type [22], and the measures has positive effect on postoperative hyponatremia [23]). . This research seeks to comprehensively investigate the incidence rate, age distribution, and severity of preoperative hyponatremia in elderly individuals with hip fractures, thereby establishing a theoretical foundation for its effective management.

## Materials and methods

### General information

Between September 2017 and December 2022, a cohort of 419 elderly individuals diagnosed with hip fractures (referred to as the fracture group) at Department of Orthopedics, The Affiliated Hospital of Chengde Medical University, were retrospectively analyzed. This group was further classified into 213 patients with femoral neck fractures and 206 with intertrochanteric femur fractures. The demographic composition included 150 males and 269 females, aged between 60 and 94 years, with an average age of 75 ± 8.74 years.

The inclusion criteria for the fracture groups were as follows: (1) Age ≥ 60 years, (2) Time from injury to admission ≤ 20 days, (3) Confirmation of unilateral femoral neck fracture or intertrochanteric femur fracture through X-ray and/or CT scan, (4) Availability of complete clinical data, and (5) Low-energy injury.

The inclusion criteria for the control groups were as follows: (1) Age ≥ 60 years, (2) Healthy elderly individuals with complete health check data.

Conversely, exclusion criteria encompassed: (1) Patients with old fractures, (2) Patients with multiple injuries, (3) Patients with multiple fractures, (4) Patients with high-energy injuries, (5) Patients with acute and chronic digestive system diseases, (6) Patients with open fractures, (7) Patients with cranial or cervical spinal cord injuries, (8) Patients who have recently or chronically used medications such as diuretics, tricyclic antidepressants, cyclophosphamide, etc., which affect water and electrolyte metabolism, and (9) Patients with pathological fractures.

### Methods

The participants were stratified into two cohorts: a fracture group and a control group, based on the presence or absence of fractures. Within the fracture group, further subgrouping was conducted based on the specific fracture location, distinguishing between femoral neck fractures and intertrochanteric femur fractures. Patients in the fracture group underwent a comprehensive biochemical panel, encompassing serum sodium levels, age, gender, and comorbid chronic diseases, within 24 h of admission. Hyponatremia was defined as a serum sodium concentration below 135 mmol/L or the lower limit of detection [24]. Subcategories of hyponatremia included mild (130–135 mmol/L), moderate (125–129 mmol/L), and severe (< 125 mmol/L).

We systematically examined variations in baseline characteristics, such as gender, age, and comorbid chronic diseases, between the groups. Additionally, we investigated the incidence rate of hyponatremia, variations in hyponatremia incidence across age groups (60–69, 70–79, ≥ 80 years), and the severity of hyponatremia. Furthermore, we compared the incidence rate and severity of hyponatremia between patients with femoral neck fractures and those with intertrochanteric femur fractures.

Approval for this study was obtained from the Ethics Committee of the Affiliated Hospital of Chengde Medical College. Informed consent was obtained from all study participants, who signed the consent forms. The findings of this research hold significant implications for understanding and managing hyponatremia in elderly patients with fractures, contributing valuable insights to clinical practice.

### Statistical analysis

Statistical analyses were performed using IBM SPSS Statistics for Windows, version 26.0 (IBM Corp., Armonk, NY, United States). Qualitative variables were presented as numbers (percentages) and were analyzed by the chi-square test. Mann-Whitney U test was used for comparing hyponatremia severity within the groups. P values less than 0.05 were considered statistically significant.

## Results

### Comparison of baseline data between the two groups

Various comorbidities were identified among the participants, including hypertension (193 cases), diabetes (89 cases), cardiovascular disease (73 cases), cerebrovascular disease (124 cases), respiratory disease (86 cases), chronic liver disease (10 cases), and chronic kidney disease (15 cases). Simultaneously, a control group consisting of 166 elderly individuals undergoing routine health checks during the same timeframe was selected. This control group comprised 67 males and 99 females, aged between 60 and 95 years, with an average age of 75.58 ± 6.58 years. Comorbidities in this control group encompassed hypertension (89 cases), diabetes (32 cases), cardiovascular disease (23 cases), cerebrovascular disease (7 cases), respiratory disease (25 cases), chronic liver disease (6 cases), and chronic kidney disease (7 cases).

The study groups exhibited no statistically significant differences in gender, age, or comorbidities, including hypertension, diabetes, cardiovascular disease, respiratory disease, chronic liver disease, and chronic kidney disease (*P* > 0.05). Notably, the fracture group demonstrated a heightened incidence of cerebrovascular disease in contrast to the control group (χ² = 44.06, *P* < 0.05). Refer to Table 1 for comprehensive information.

**Table 1:** Comparison of general conditions and comorbid diseases among the study participants

		Fracture group	Control group	χ² value	*P* value
Gender	Male	150	67	1.060	0.303
	Female	269	99		
Age	≤ 74 years	206	75	0.756	0.385
	> 75 years	213	91		
Hypertension		193(46.06%)	89(53.61%)	2.716	0.099
Diabetes		89(21.24%)	32(19.28%)	0.280	0.597
Cardiovascular disease		73(33.33%)	23(13.86%)	1.103	0.294
Cerebrovascular disease		124(29.59%)	7(4.22%)	44.060	0.000
Respiratory disease		86(20.53%)	25(15.06%)	2.310	0.129
Chronic liver disease		10(2.39%)	6(3.61%)	0.291	0.589
Chronic kidney disease		15(3.58%)	7(4.22%)	0.133	0.715

The incidence rate of cerebrovascular disease in the fracture group patients was higher than that in the control group person (χ² = 44.06, *P* < 0.05). There were no statistically significant differences in baseline characteristics and other concomitant chronic diseases between the two groups (*P* > 0.05). See Table 1.

### Investigating the occurrence of hyponatremia in the two groups

The prevalence of hyponatremia within the fracture group was 22.20% (93 out of 419), whereas the control group exhibited a notably lower rate of 1.81% (3 out of 166). Statistical analysis revealed a significant disparity in the incidence of hyponatremia between the fracture and control groups (χ² = 36.030, *P* < 0.05), as delineated in Table 2.

**Table 2:** Comparison of incidence rate of hyponatremia between groups

	Fracture group	Control group	χ² value	*P* value
Hyponatremia	93(22.20%)	3(1.81%)	36.030	0.000

In the fracture group of 419 patients, 93 (22.20%) were diagnosed with hyponatremia at admission. In the control group of 166 elderly individuals, 3(1.81%) were diagnosed with hyponatremia. The prevalence of hyponatremia in fracture group patients was statistically significantly higher than that in the control group (χ² = 36.030, *P* < 0.05) See Table 2.

### Examining the occurrence rate of hyponatremia among various age categories in the comparative analysis of the two groups

In the fracture group, the incidence of hyponatremia in patients aged 60–69, 70–79, and ≥ 80 years were 11.85% (16/135), 20.93% (27/129), and 32.26% (50/155). In comparison, the control group exhibited rates of 0.00% (0/26), 1.09% (1/92), and 4.17% (2/48) for the same age groups, respectively. Notably, within the 70–79 and ≥ 80 years age brackets, the fracture group demonstrated significantly higher hyponatremia incidence rates when compared to the control group (χ² = 19.111, *P* < 0.05; χ² = 15.179, *P* < 0.05). Conversely, there was no statistically significant difference in hyponatremia incidence between the fracture and control groups in the 60–69 age group (χ² = 2.225, *P* > 0.05). Detailed information is available in Table 3.

**Table 3:** Comparison of Incidence Rate of Hyponatremia Across Different Age Groups

	60–69 years old	70–79 years old	≥ 80 years old
Fracture group	16/135	27/129	50/155
Control group	0/26	1/92	2/48
χ² value	2.225	19.111	15.179
*P* value	0.136	0.000	0.000

### Extent of hyponatremia severity in the two groups

Within the fracture group, the incidence rates of non-hyponatremia and mild, moderate, and severe hyponatremia stood at 77.80% (326 out of 419), 17.90% (75 out of 419), 3.10% (13 out of 419), and 1.19% (5 out of 419), respectively. In contrast, the control group exhibited rates of 98.19% (163 out of 166), 1.81% (3 out of 166), 0.00% (0 out of 166), and 0.00% (0 out of 166) for the corresponding categories, respectively. Notably, the severity of hyponatremia was significantly elevated in the fracture group, as evidenced by a Z-score of — 6.005(*P* < 0.05). Refer to Table 4 for comprehensive results of these findings.

**Table 4:** Comparison of Severity of Hyponatremia Between Groups

	Non-hyponatremia	Mild hyponatremia	Moderate hyponatremia	Severe hyponatremia
Fracture group	326	75	13	5
Control group	163	3	0	0
Z value	-6.005			
*P* value	0.000			

The incidence of non-hyponatremia, mild, moderate and severe hyponatremia in fracture group patients was 77.80% (326/419), 17.90% (75/419), 3.10% (13/419) and 1.19% (5/419). The incidence of non-hyponatremia, mild, moderate and severe hyponatremia in the control group person was 98.19% (163/166), 1.81% (3/166), 0.00% (0/166) and 0.00% (0/166). The severity of hyponatremia in the fracture group was significantly higher than that in the control group (Z= -6.005, *P* < 0.05). See Table 4.

### Examining the occurrence rate and intensity of hyponatremia in patients with intertrochanteric femur fractures versus those with femoral neck fractures

The prevalence of hyponatremia in patients with intertrochanteric femur fractures stood at 27.18% (56 out of 206), surpassing the rate observed in the femoral neck fracture group, which was 17.37% (37 out of 213). Notably, the intertrochanteric femur fracture group exhibited a significantly higher incidence of hyponatremia (χ² = 5.840, *P* < 0.05) as detailed in Table 5. Within the intertrochanteric femur fracture cohort, 72.82% (150 out of 206) did not develop hyponatremia, while 22.82% (47 out of 206), 2.43% (5 out of 206), and 1.94% (4 out of 206) experienced mild, moderate, and severe hyponatremia, respectively. In contrast, the femoral neck fracture group displayed prevalence rates of 82.63% (176 out of 213), 13.15% (28 out of 213), 3.76% (8 out of 213), and 0.47% (1 out of 213) for non-hyponatremia and mild, moderate, and severe hyponatremia. Significantly higher severity of hyponatremia was evident in the intertrochanteric femur fracture group (Z= -2.321, *P* < 0.05). See Table 6 for details.

**Table 5:** Incidence Rate of Hyponatremia in Elderly Patients with Hip Fractures

		Femoral neck fracture group	Intertrochanteric fracture group	χ² value	*P* value
Hyponatremia	Yes	37	56	5.840	0.016
	No	176	150		

**Table 6:** Severity of Hyponatremia in Elderly Patients with Hip Fractures

	Non-hyponatremia	Mild hyponatremia	Moderate hyponatremia	Severe hyponatremia
Femoral neck fracture group	176	28	8	1
Intertrochanteric fracture group	150	47	5	4
Z value	-2.321			
*P* value	0.020			

The incidence of hyponatremia was 27.18% (56/206) in the intertrochanteric fracture group and 17.37% (37/213) in the femoral neck fracture group. The incidence of hyponatremia in the intertrochanteric fracture group was significantly higher than that in the femoral neck fracture group (χ²=5.840, *P* < 0.05) (see Table 5). The incidence of non-hyponatremia, mild, moderate and severe hyponatremia in patients with intertrochanteric fracture was 72.82% (150/206), 22.82% (47/206), 2.43% (5/206) and 1.94% (4/206). The incidence of non-hyponatremia, mild, moderate, and severe hyponatremia in femoral neck fracture patients was 82.63% (176/213), 13.15% (28/213), 3.76% (8/213) and 0.47% (1/213). The severity of hyponatremia in the intertrochanteric fracture group was significantly higher than that in the femoral neck fracture group (Z= -2.321, *P* < 0.05) (see Table 6).

## Discussion

### Comparative analysis of baseline data between the two groups

The results of this study demonstrated a heightened prevalence of cerebrovascular disease in the fracture group compared to the control group, attributable to several factors: (1) Patients with cerebrovascular diseases are often accompanied by hypertension [25]. The incidence of hyponatremia in patients with cerebrovascular diseases is high due to dietary restriction and other reasons [26]. (2) Certain individuals with cerebrovascular disease experience sensory and motor impairments, amplifying the susceptibility to falls and consequent injuries [27, 28]; (3) Prolonged cerebrovascular disease may lead to disuse osteoporosis among patients, thereby escalating the likelihood of fractures [29, 30]; and (4) A majority of patients in the fracture group underwent routine cranial CT scans upon admission, facilitating the detection of even latent or asymptomatic cerebrovascular diseases.

### Etiology of hyponatremia among individuals with fractures

There was a heightened incidence and severity of hyponatremia in the fracture group compared to the control group, with the incidence rate escalating with age. The majority of elderly hip fractures result from low-energy injuries against an osteoporotic background. Pre-existing hyponatremia and osteoporosis increase vulnerability to fractures. The following key factors were identified: (1) Mild hyponatremia, which shows symptoms such as dizziness, fatigue, weakness, nausea, vomiting, apathy, sleepiness, slow reaction, and lack of concentration. Moderate hyponatremia may manifest as cognitive impairment, orientation disturbance, and an unsteady gait. Severe hyponatremia poses the risk of coma, seizures, and other cerebral disorders, potentially becoming life-threatening [31, 32]. Consequently, individuals with hyponatremia are more predisposed to falls and fractures. (2) Other studies [33, 34] confirm the association between hyponatremia, bone loss and osteoporosis. (3) The fracture group, primarily composed of elderly individuals, frequently encountered compromised digestive and absorption functions, along with the prevalence of multiple chronic diseases, affecting their regular dietary intake.

### Etiology of hyponatremia in individuals suffering from intertrochanteric femur fractures

There was a higher incidence rate of hyponatremia (27.18%) in individuals with intertrochanteric femur fractures compared to those with femoral neck fractures (17.37%). Furthermore, the severity of hyponatremia was more pronounced in patients with intertrochanteric femur fractures, attributed to several factors: (1) Intertrochanteric femur fractures, being extra-particular, are associated with substantial occult blood loss, leading to blood dilution [35]. (2) These fractures often entail larger displacements and more intense pain, potentially impacting the patient’s dietary habits and resulting in reduced sodium intake. (3) Patients with intertrochanteric femur fractures typically exhibit advanced age, severe osteoporosis, and consequently, a heightened degree of hyponatremia.

### Correlation between hyponatremia and hip fractures in the elderly

Fractures in the elderly, particularly hip fractures, often result from minor external forces acting upon a backdrop of osteoporosis. The prolonged presence of hyponatremia prompts the leaching of sodium ions from bones into the bloodstream to sustain normal metabolic functions, thereby fostering the development of osteoporosis. Furthermore, animal studies [36] have demonstrated that hyponatremia augments osteoclastic bone resorption, liberating stored sodium from bones and thereby contributing to the progression of osteoporosis. Consequently, the presence of hyponatremia serves as an indicative marker of the extent of osteoporosis in patients. The occurrence of preoperative hyponatremia in elderly individuals with hip fractures not only directly signifies the severity of hyponatremia but also serves as an indicator of the degree of osteoporosis, along with the associated risk of falls and fractures.

Recent research [37] has conclusively established that elderly patients with hip fractures and coexisting hyponatremia exhibit a heightened incidence of perioperative complications, prolonged hospitalization, and elevated mortality rates compared to their counterparts with normal serum sodium levels. Hence, the timely identification and correction of hyponatremia prior to surgery can significantly contribute to the recovery and prognosis of patients.

### Study strengths and limitations

This study comprehensively investigates the incidence, age distribution, and intensity of preoperative hyponatremia among elderly individuals with hip fractures. The way to prevent hyponatremia is to monitor serum sodium and supplement sodium as needed regularly. Regular monitoring of hyponatremia is becoming increasingly important, so a simple and convenient method for monitoring hyponatremia deserves further study. However, the data on postoperative hyponatremia in elderly hip fracture patients were lacking in this study, and the research was a retrospective, single-center study.

## Conclusion

In conclusion, our research underscores a substantial incidence of preoperative hyponatremia among elderly individuals with hip fractures, predominantly manifesting in mild to moderate forms and demonstrating an escalating prevalence with advancing age. Particularly noteworthy is the heightened incidence and severity of hyponatremia observed in patients with intertrochanteric femur fractures, necessitating careful consideration by healthcare practitioners. Furthermore, the results of our study suggest a potential link between hyponatremia and the etiology of hip fractures in the elderly, positing that corrective measures for hyponatremia could serve as a viable strategy for preventing such fractures and ultimately improving the overall prognosis for elderly patients grappling with hip fractures.

## Data Availability

No datasets were generated or analysed during the current study.
